# Sepsis and Acute Kidney Injury: A Review Focusing on the Bidirectional Interplay

**DOI:** 10.3390/ijms23169159

**Published:** 2022-08-15

**Authors:** Yu-Ming Chang, Yu-Ting Chou, Wei-Chih Kan, Chih-Chung Shiao

**Affiliations:** 1 Division of Nephrology, Department of Internal Medicine, Camillian Saint Mary’s Hospital Luodong, Yilan 26546, Taiwan; 2 Department of Internal Medicine, National Taiwan University Hospital, Taipei 100225, Taiwan; 3 Department of Nephrology, Department of Internal Medicine, Chi Mei Medical Center, Tainan 71004, Taiwan; 4 Department of Biological Science and Technology, Chung Hwa University of Medical Technology, Tainan 71703, Taiwan; 5 Saint Mary’s Junior College of Medicine, Nursing and Management, Yilan 26546, Taiwan

**Keywords:** acute kidney injury, infection, sepsis, pathophysiology, sepsis-associated acute kidney injury

## Abstract

Although sepsis and acute kidney injury (AKI) have a bidirectional interplay, the pathophysiological mechanisms between AKI and sepsis are not clarified and worthy of a comprehensive and updated review. The primary pathophysiology of sepsis-associated AKI (SA-AKI) includes inflammatory cascade, macrovascular and microvascular dysfunction, cell cycle arrest, and apoptosis. The pathophysiology of sepsis following AKI contains fluid overload, hyperinflammatory state, immunosuppression, and infection associated with kidney replacement therapy and catheter cannulation. The preventive strategies for SA-AKI are non-specific, mainly focusing on infection control and preventing further kidney insults. On the other hand, the preventive strategies for sepsis following AKI might focus on decreasing some metabolites, cytokines, or molecules harmful to our immunity, supplementing vitamin D3 for its immunomodulation effect, and avoiding fluid overload and unnecessary catheter cannulation. To date, several limitations persistently prohibit the understanding of the bidirectional pathophysiologies. Conducting studies, such as the Kidney Precision Medicine Project, to investigate human kidney tissue and establishing parameters or scores better to determine the occurrence timing of sepsis and AKI and the definition of SA-AKI might be the prospects to unveil the mystery and improve the prognoses of AKI patients.

## 1. Introduction

Acute kidney injury (AKI) is an essential but complex syndrome that results from heterogeneous mechanisms and carries considerable morbidity and mortality [1,2]. AKI occurs in 10%–15% of hospitalized patients and 50% in the intensive care unit (ICU) setting [3,4]. AKI is recognized as a decrease in glomerular filtration rate (GFR), and the contemporarily accepted system for definition and classification of AKI is the Kidney Disease Improving Global Outcomes (KDIGO) Clinical Practice Guideline for AKI 2012 [5], which is based on serum creatinine, GFR, and urine output. Although the KDIGO 2012 AKI guideline has many limitations, it could not be replaced by any other AKI definition/classification/staging systems [6]. Although there has been some advancement in AKI therapies, including kidney replacement therapy (KRT), the improvement in AKI patients’ outcomes has been limited over the decades [7,8]. The disappointing results call for a strategy to prevent the occurrence and progression of AKI at an earlier stage. For this purpose, a crucial step is to obtain a more profound and clear understanding of the association between AKI and other underlying clinical illness or factors that cause or precipitate AKI occurrence [9,10].

Sepsis, a life-threatening organ dysfunction resulting from a dysregulated host response to infection, causes heavy healthcare and economic burdens worldwide [4,11,12,13]. At the same time, sepsis is a primary and crucial precipitating factor of AKI in critically ill patients. It is known that sepsis increases the risk of AKI development [11], and an increasing body of evidence reveals higher risks of infection or sepsis following AKI [12]. Due to the close relationship between AKI and sepsis, some investigators suggested taking AKI as an early sign of sepsis [13]. As to the molecular-level aspect, existing evidence, including our previous work, has demonstrated procalcitonin’s optimal ability to detect sepsis and AKI [14,15]. Since procalcitonin is an infection biomarker, these findings indicate the complicated association between sepsis and AKI. However, the pathophysiological mechanisms between AKI and sepsis are not clearly understood and are worthy of a comprehensive and updated review.

The current narrative review focused on the bidirectional pathophysiologies between AKI and sepsis. We extended sepsis to a broader field to include severe infection to provide a more comprehensive review. Although most proposed theories are based on animal models, this knowledge helps physicians and researchers understand the underlying pathophysiology crucial for new perspectives to improve the patients’ prognoses of these populations.

## 2. Epidemiology

Although it is well known that sepsis increases the subsequent AKI risks, the incidence of sepsis-associated AKI (SA-AKI) has not been well determined [11]. The incidence rates of AKI among septic patients were reported as 54% in a multicenter prospective cohort study enrolling critically ill patients from 24 European countries [16] and 47.1% in a multicenter retrospective cohort study enrolling hospitalized patients across China [17]. According to the observation that AKI occurs in roughly one to two out of three septic patients, the SA-AKI has involved six to eleven million cases [11,18]. Besides, sepsis is also the leading cause of death in AKI patients [19].

On the other hand, AKI is associated with a higher risk of de novo infections and the progression of sepsis. Among the cardiac surgical patients, postoperative AKI influenced the risk of severe infections in a retrospective analysis of 24,660 patients [20], and even mild AKI was independently associated with postoperative infection [21]. Besides surgical patients, a higher infection risk following AKI was also noticed in non-surgical patients [12,22,23,24]. In a propensity score-matched cohort study studying hospitalized patients, Griffin BR et al. found that compared to the patients without AKI, those with recovered AKI whose creatinine returned to baseline were still associated with a 4.5-fold increased odds ratio for infection within 30 days following discharge [22]. The association between AKI and subsequent infection persistently exists in a nationwide population-based cohort study. A previous work of our study group demonstrated that patients with severe AKI requiring KRT had an approximately 3-fold higher risk of developing severe sepsis than the non-AKI group (6.84 versus 2.32 per 100 person-years) in the index hospitalization. The hazard ratios for severe sepsis in the comparisons between the KRT-requiring AKI group and non-AKI group were 2.05–3.44 in different adjustment models with various follow-up periods of the multivariate analyses. The subgroup analysis found that even the patients who recovered from the KRT-requiring AKI had significantly higher hazard ratios (1.61–1.68) of developing severe sepsis than those without KRT-requiring AKI [12]. Another previous work of our team also observed an increased risk of active tuberculosis following KRT-requiring AKI in the Taiwan National Health Insurance database. This association was also noted even in patients weaned from dialysis, suggesting that immune dysfunction in AKI is not limited in the short term [23]. A retrospective cohort study showed that the three most frequent infections in critical patients with AKI were pneumonia (54.3%), intra-abdominal infection (11.9%), and urinary tract infections (9.7%) [24].

## 3. Pathophysiology of SA-AKI

The pathophysiology of SA-AKI remains unclear, although scientists have made considerable effort in this field. Most current theories are based on animal models or autopsy results [11]; thus, the interpretation and application of these findings should be made cautiously. In an earlier era, renal hypoperfusion and associated ischemia were considered the main culprits of SA-AKI, but animal experiments have not entirely supported this traditional concept.

**Figure 1:**
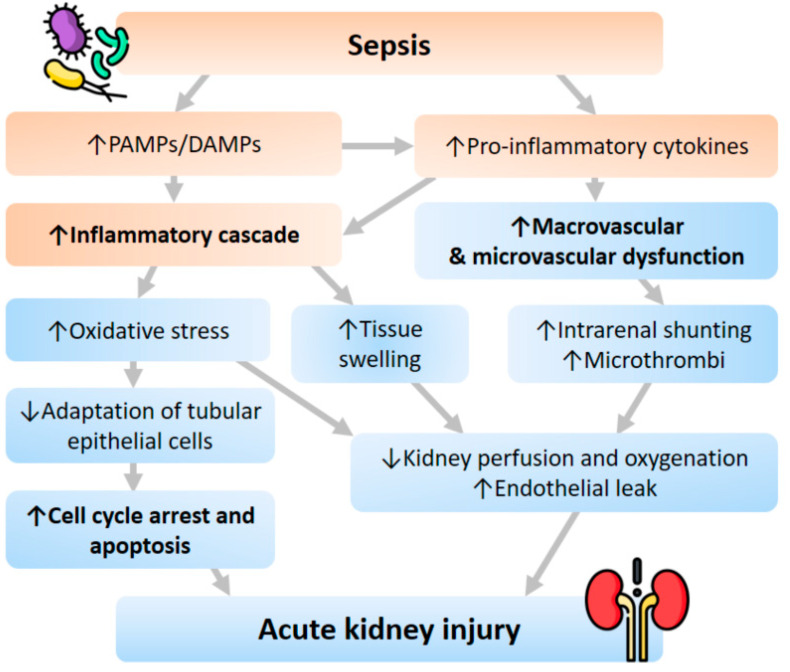
Pathophysiology of AKI following sepsis. Abbreviations: DAMPs, damage-associated molecular patterns; PAMPs, pathogen-associated molecular patterns.

Several animal studies demonstrated that the subjects with Gram-negative bacteremia had significantly increased renal blood flow compared with the controlled group. In histology findings, the degree of tubular injury was mild, and there was no significant difference between the sepsis and non-sepsis groups [25,26,27]. Moreover, an animal model did not reveal a significant correlation between early SA-AKI and histopathologic lesions on renal biopsy [25]. Therefore, instead of the traditional concept, inflammatory cascade, macrovascular and microvascular dysfunction, and cell response abnormality are now believed to be the three cornerstones of the underlying pathophysiological mechanism of SA-AKI (Figure 1).

### 3.1. Inflammatory Cascade

In the state of sepsis, inflammatory mediatory molecules such as pathogen-associated molecular patterns (PAMPs) and damage-associated molecular patterns (DAMPs) are released into the intravascular space and bind to receptors such as Toll-like receptors (TLRs) on the surface of immune cells. This reaction subsequently initiates a sequence of the signal cascade, producing and releasing pro-inflammatory cytokines. Besides, renal tubular epithelial cells also have expressed TLRs, especially TLR-2 and TLR-4. Thus, a similar pathway will be activated once PAMPs or DAMPs are filtered through the glomerulus, leading to increased oxidative stress, production of reactive oxygen species, and mitochondrial injury [11,28,29].

### 3.2. Macrovascular and Microvascular Dysfunction

At the same time, efferent arteriolar vasodilation and intrarenal shunting contribute to macrovascular dysfunction. The macrovascular dysfunction diverts the renal blood flow from the medulla to the cortex, causing decreased perfusion and oxygenation of the medulla, further worsening kidney function [30]. As for the microvascular system, elevated pro-inflammatory cytokines and activated leukocytes in sepsis might lead to microthrombi formation in renal capillaries [31]. The microthrombi formation results in decreased blood flow and diffusion to the inflamed and edematous tissue [32]. These vascular dysfunctions also cause the production of reactive oxygen species that further damage the epithelial barrier that ends up in an endothelial leak [33,34].

### 3.3. Cell Cycle Arrest and Apoptosis

To date, different theories have been proposed to explain the metabolic adaptation that tubular epithelial cells undergo in sepsis. The fundamental concepts of these theories include optimizing energy utilization, priority preserving vital cell functions, and avoiding cell death. During sepsis, the cell adaption downregulates aerobic glycolysis and increases oxidative phosphorylation to improve survival and make the host less vulnerable to developing AKI [35,36,37]. Since cell replication is a very energy-consuming process, several checkpoints are the sentinels to evaluate whether the cell has sufficient energy to undergo replication during the cell cycle. If the energy is deemed insufficient, the cell will proceed into cell cycle arrest to avoid energy failure and apoptosis. Any delay or inadequacy of this rescue action finally results in cell death. Both cell cycle arrest and apoptosis play essential roles in the initiation of SA-AKI.

Excellent examples to support the primary roles of cell cycle arrest and apoptosis in SA-AKI development are the two emerging biomarkers, the tissue inhibitor of metalloproteinase-2 (TIMP-2) and insulin-like growth factor-binding protein-7 (IGFBP-7). These two molecules are basically cell cycle arrest biomarkers, but they have optimal predictive ability for SA-AKI [11,25].

## 4. Pathophysiology of Sepsis Following AKI

**Figure 2:**
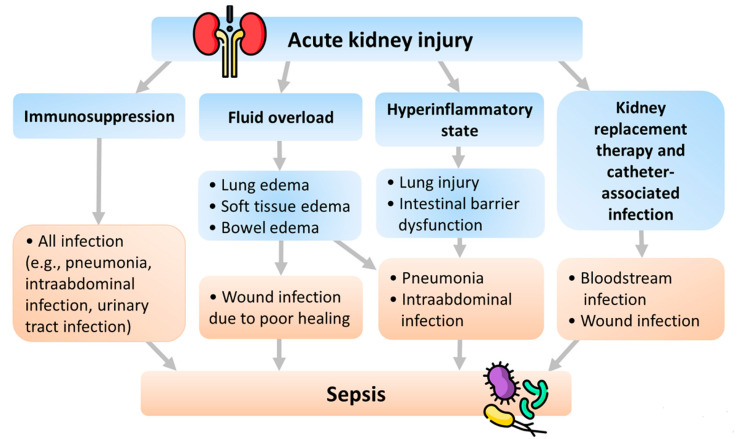
Pathophysiology of sepsis following AKI.

Several pathophysiologies of sepsis following AKI have been proposed. We summarize these pathophysiologies in Figure 2 and explain them below (Figure 2). Pneumonia is the most frequent infection that results from volume overload and inflammation in AKI patients. Moreover, there is a close and complex association between AKI, immune dysregulation, pneumonia, and lung injury. Therefore, we also include lung injury in addition to pneumonia in the following sections to provide readers with a more comprehensive understanding.

### 4.1. Fluid Overload

Fluid overload is a common complication of oliguria or anuria in AKI that presents with tissue edema. Soft tissue edema causes poor tissue healing and wound infection [38]. Bowel edema impairs barriers to infection, allowing intestinal bacteria translocation and leading to the development of intraabdominal infection or even sepsis [38]. Besides, lung edema occurs due to fluid accumulation, increased vascular permeability, and down-regulation of the sodium-potassium pump and aquaporin in AKI. The reduced aquaporin activity resulting from AKI [39] also predisposes ventilator-induced lung injury [40]. Lung edema, along with another complication of fluid overload, pleural effusion, exacerbates subsequent lung atelectasis, pneumonia, and empyema [38].

### 4.2. Hyperinflammatory State

AKI presents a hyperinflammatory state with reduced cytokine clearance and increased systemic cytokine levels, such as interleukin (IL)-17A, IL-6, IL-8, IL-1 beta, IL-12, and tumor necrosis factor (TNF)-alpha [41,42,43,44]. During AKI, the increased IL-17A attributes to Paneth cells in the small intestines and results in neutrophil influx, T cell activation, IL-6 overproduction, hepatic injury, and intestinal barrier disruption. IL-1 beta, IL-2, IL-6, IL-8, IL-1 beta, IL-12, TNF-alpha, and their receptors escalate early after AKI and lead to pro-inflammatory, neutrophil activation, endothelial cell apoptosis, and endothelial dysfunction in animal models [41,42,43,44]. Besides, some other plasma cytokines (e.g., keratinocyte-derived chemokine and Granulocyte-macrophage colony-stimulating factor) are generated during AKI. Nevertheless, although the plasma IL-6 is elevated, the monocyte cytokine production of IL-1beta, TNF-alpha, and IL-6 is impaired in critically ill AKI patients. This cytokine pattern in AKI patients is more similar to critically ill patients without AKI than those non-critically ill patients with chronic kidney disease (CKD) [45]. This presentation is also consistent with the finding that the AKI-associated hyperinflammatory state suppresses the immune system function and impairs the clearance of infection [46,47].

Elevated serum inflammatory biomarkers, such as IL-6, plasminogen activator inhibitor-1, and TNF-alpha, have essential roles in AKI and pneumonia. The circulating cytokine levels are higher in the patients with both AKI and pneumonia than in those has only pneumonia, suggesting the influence of AKI on cytokine is independent of infection. Furthermore, higher immune response in patients with AKI and even mild pneumonia links with increased mortality risk [48]. IL-6 induces increased pulmonary chemokine (C-X-C motif) ligand 1 (CXCL1) expression in AKI, promoting lung neutrophil infiltration. On the contrary, blockade of the CXCL1 signal reduces neutrophil infiltration in the lung, and IL-6 knockout mice are resistant to lung injury following ischemic AKI [49]. Increased neutrophil infiltration, vascular permeability, salt and water transporters dysregulation, and inflammatory cytokines are crucially associated with AKI-induced lung injury [50]. Besides neutrophils, T lymphocytes and macrophages also mediate lung injury during AKI. T lymphocyte administration induces pulmonary cellular apoptosis and lung microvascular barrier dysfunction, whereas macrophage mediates increased pulmonary vascular permeability via chemokine production in the experimental AKI model. Furthermore, AKI causes lung injury by inducing a pro-inflammatory and proapoptotic lung endothelial cell response with TNF receptor 1-dependent caspase activation, programmed cell death, and microvascular barrier dysfunction [51,52,53].

TLR-4 is essential in recognizing pathogens, such as lipopolysaccharide, heparan sulfate, heat shock proteins, and high-mobility group box protein B1 (HMGB1). TLR-4 actives humoral and cellular adaptive immune responses and is associated with neutrophil infiltration, increased neutrophil elastase activity, and vascular permeability in the lung [54]. HMGB1, a pro-inflammatory cytokine released from apoptotic cells, interacts with TLR-4 on target cells and activates nuclear factor kappa B and immunostimulatory responses [55]. In the ischemic AKI model, HMGB1 blockade reduced pulmonary neutrophil infiltration independent from TLR-4. These observations suggested that the TLR-4-HMGB1 pathway contributes to AKI-induced lung injury and has variable effects on different types of AKI [56].

### 4.3. Immunosuppression

Although AKI causes a pro-inflammatory response under general conditions, AKI may attenuate the neutrophil’s inflammatory effects under inflammatory states. Several reports found that neutrophil recruitment into inflamed organs decreased significantly during AKI, reducing the bacterial killing and promoting infection [57,58,59]. In animal models, mice with concomitant pneumonia and AKI had milder pulmonary inflammation than those with pneumonia only because AKI diminishes neutrophil recruitment into the lung during severe lung injury [57]. As in human investigation, the neutrophil function is more intensely suppressed in patients with AKI and sepsis than in patients with sepsis alone, suggesting that AKI interferes with neutrophil function [58].

A typical process of neutrophil recruitment includes capture, rolling, slow-rolling, firm adhesion, and transmigration. AKI impedes neutrophils’ slowing rolling and transmigration by abating E-selectin/intercellular adhesion molecule-1 and P-selectin/intercellular adhesion molecule-1. The selectin-mediated slow leukocyte rolling is inhibited by reduced phosphorylation of spleen tyrosine kinase, Akt, phospholipase C-γ2, and p38 mitogen-activated protein kinases [59,60]. The F-actin formation is a crucial process of neutrophil migration. AKI impedes neutrophil migration by impairing F-actin formation by interfering with the phosphatidylinositol 3-kinase-γ and phospholipase C-γ2-dependent pathways [57,58].

**Figure 3:**
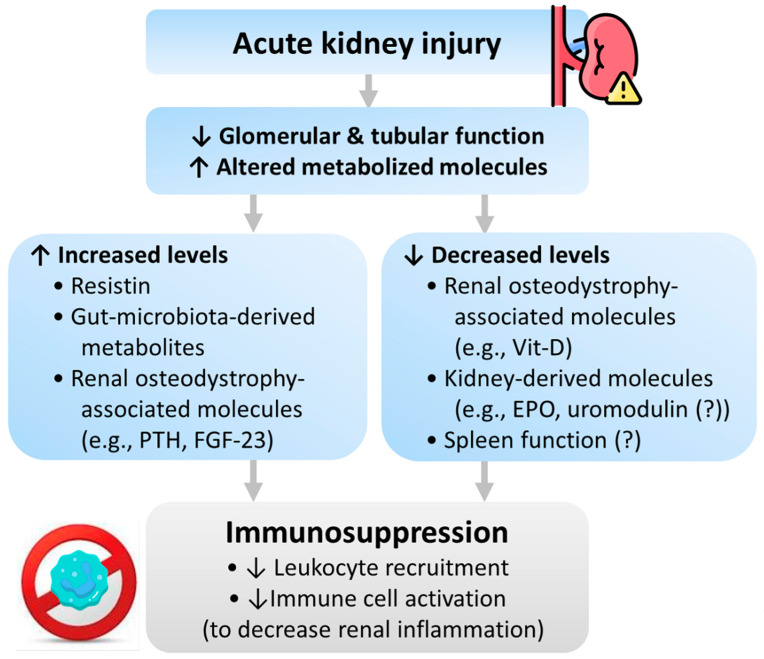
Pathophysiology of immunosuppression following AKI. Abbreviations: EPO, erythropoietin; FGF-23, fibroblast growth factor-23; PTH, parathyroid hormone; Vit-D, vitamin D.

Besides, impaired monocyte function has also occurred in AKI. This kind of immunosuppression is more similar to patients with systemic inflammatory response syndrome rather than patients with CKD or end-stage kidney disease, suggesting that inflammation rather than kidney function is a significant determinant factor of immune dysregulation in AKI [45]. The AKI-associated immunosuppression might result from uremic toxins and abnormal metabolites molecules secondary to AKI. We summarize these pathophysiologies in Figure 3.

#### 4.3.1. Resistin

Resistin is a 12-kDa uremic toxin and pro-inflammatory cytokine. Increased serum resistin concentrations positively correlate with sepsis severity [61]. The serum resistin level is less than 20 ng/mL in healthy individuals, which might increase to 30–40 ng/mL in end-stage kidney disease patients and further increase to higher than 100 ng/mL in patients with septic shock and AKI [62]. Under high concentrations (more than 20 ng/mL), resistin blocks phosphorylation of the phosphatidylinositol 3-kinase pathway, and subsequently inhibits neutrophil chemotaxis, neutrophil migration, and bacterial killing ability. Besides, resistin impairs phosphoinositide-dependent kinase 1, an essential protein for actin polymerization. Since actin polymerization is responsible for reactive oxygen species and neutrophil migration, resistin may contribute to the disturbed immune response by diminishing neutrophil migration [58,63]. The above mechanism is supported by a study showing that resistin causes neutrophil dysfunction, resembling AKI-associated neutrophil dysfunction [58].

#### 4.3.2. Gut Microbiota-Derived Metabolites

AKI causes urea accumulation and increased urea influx into the gut, where urea is converted into ammonia that disrupts the epithelial tight junction of the gut and aggravates bacterial translocation [64]. During AKI, gut dysbiosis develops via both innate immunity (macrophages and neutrophils) and adaptive immunity (T-helper 17 cell) pathways [65]. Gut dysbiosis decreases short-chain fatty acids and increases gut-derived uremic toxins (e.g., indoxyl sulfate and p-cresyl sulfate), which alters immune homeostasis and further exacerbates AKI [65,66]. Besides, gut dysbiosis also contributes to intestinal inflammation and leaky gut [66]. The neutrophils, pro-inflammatory macrophages, and T-helper 17 cells accumulated in the gut impair barrier integrity and enhance bacterial translocation [67], and the increased plasma TNF-α, IL-17A, and IL-6 lead to endothelial apoptosis and epithelial necrosis in the small intestine [68,69]. All factors mentioned above contribute to immunosuppression and consequent infection.

#### 4.3.3. Renal Osteodystrophy-Associated Molecules

Vitamin D, parathyroid hormone (PTH), and fibroblast growth factor-23 (FGF-23) are biomarkers of renal osteodystrophy. Their abnormal homeostases in AKI and CKD also influence immune function. The active vitamin D metabolite, 1,25-dihydroxy-vitamin D3 (calcitriol), is converted from vitamin D by the kidney; thus, vitamin D production is decreased in AKI. Vitamin D has been shown to promote macrophage phagocytosis, regulate monocyte immune function, and diminish inflammatory cytokine production. The regulation of monocyte function is mainly through the stimulation of the cathelicidin, an endogenous anti-microbial peptide produced by macrophages and neutrophils [70]. Besides, calcitriol markedly enhances tight junctions and decreases susceptibility to mucosal barrier damage and subsequent infection [71]. Clinically, a systemic review and meta-analysis revealed that vitamin D deficiency increases the risks of severe infections and mortality of critically ill patients [72].

Hyperparathyroidism, often occurring in AKI and CKD settings, is associated with abnormal immune function. High PTH levels inhibit T lymphocyte transformation and CD4/CD8 ratio and produce dose-dependent inhibition of B lymphocyte proliferation and immunoglobulin production. Besides, high PTH also influences polymorphonuclear leukocytes by inhibiting migration, phagocytosis, bactericidal activity, and chemotaxis [73,74].

FGF-23 is a bone-derived hormone whose serum levels increase in AKI and CKD [75]. FGF-23 impairs immune functions through direct interactions with myeloid cells, including macrophages and polymorphonuclear leukocytes. In murine kidney injury models, FGF-23 influences leukocyte recruitment and host defense, which are restored after FGF-23 neutralization [76]. Besides, FGF23 indirectly increases inflammation and infection by suppressing vitamin D production in the kidney’s proximal tubule [77]. As a result, a higher FGF-23 concentration is associated with increased infection risk in patients irrespective of kidney disease [78,79].

#### 4.3.4. Kidney-Derived Molecules

Erythropoietin (EPO) and uromodulin are kidney-derived molecules that influence macrophage and sepsis progression. Experiments have shown that EPO level increases within the first 48 h of AKI and then drops progressively [80]. EPO benefits immunomodulation by normalizing activated CD4(+) T lymphocytes and their proliferative capacity, hastening efferocytosis, and suppressing inflammatory gene expression [18,80]. Therefore, decreased EPO levels in AKI might be associated with immunosuppression.

Uromodulin (also known as Tamm–Horsfall protein) is a high-molecular-weight polymer yielded by the kidney and excreted into the urine [81]. The polymeric uromodulin prevents bacteria from attachment to the urothelial surface. Meanwhile, uromodulin executes its immunomodulatory role by regulating macrophage number, phagocytic function, and neutrophil production [82]. Experimental studies found that uromodulin knockout mice had higher bladder and urinary tract infection risks [83,84]. Increased interstitial presence of uromodulin negatively regulates the inflammatory response in the murine’s proximal tubules and hastens kidney recovery, suggesting uromodulin as a prognostic biomarker of recovery from AKI [85]. Another experiment disclosed that circulating uromodulin dropped after AKI, which was associated with an increase in systemic reactive oxygen species. These findings suggested that uromodulin plays a crucial role in systemic oxidative stress and explained how uromodulin deficiency is associated with unfavorable outcomes [86]. As in humans, elevated serum or urinary uromodulin levels are beneficial against kidney function decline, cardiovascular events, and overall mortality [87,88,89].

#### 4.3.5. Kidney and Spleen Interactions

The spleen is a component of the reticuloendothelial system accountable for host defense. Splenic IL-10 downregulates the pro-inflammatory response following ischemic AKI. Patients with asplenia have a higher risk for fulminant infection. In subjects with AKI, splenectomy exacerbates lung injury and pneumonia. In mice with sepsis and AKI, splenocyte apoptosis causes a vicious cycle of sepsis and spleen dysfunction [90,91], indicating the association between AKI, spleen dysfunction, and sepsis.

### 4.4. KRT and Catheter-Associated Infection

Critically ill patients with AKI treated with KRT are more susceptible to a nosocomial bloodstream infection, most commonly caused by Gram-positive species [92]. Similar findings were also observed in critically ill children treated with CKRT, demonstrating that more than four days of CKRT increased the risk of infection [93].

## 5. Potentially Preventive Strategies

The preventive strategies for SA-AKI are challenging and non-specific, mainly focusing on infection control and prevention of further kidney injury secondary to other problems. These strategies are summarized below. First, promptly prescribe proper antibiotics to treat infection and sepsis. Second, prescribe adequate hydration and vasopressor, with indication, to maintain a mean arterial blood pressure >65 mmHg and renal perfusion and autoregulation. Third, choose balanced crystalloids rather than 0.9% saline as intravenous fluid. The use of balanced crystalloids plays a protective role against major kidney adverse events, and the protective effect of balanced crystalloids is more significant in septic patients than in those without sepsis [94,95]. Fourth, avoid nephrotoxic agents in patients with high AKI risk. Some nephrotoxic agents, such as aminoglycosides and vancomycin, especially when in combination with piperacillin-tazobactam and amphotericin B, should be used with great caution. Besides, contrast-enhanced imaging studies should also be weighed carefully beforehand. Fifth, apply the KDIGO bundle to septic patients as possible. The KDIGO bundle is a package of preventive measures proposed by the KDIGO guideline. The bundle’s effect on reducing AKI occurrence and severity in the high-risk postoperative septic patient is under evaluation by a controlled, prospective, randomized clinical trial started in January 2020 (ClinicalTrials.gov, NCT04222361). Sixth, keep high awareness of “abdominal compartment syndrome” since high intra-abdominal pressure is a known deteriorating factor of SA-AKI, especially after trauma, surgery, or fluid resuscitation [96]. Although there is no consensus on whether early decompression or administration of diuretics helps prevent AKI, it is still worth attention to eliminate this potent trigger [97] (Table 1).

On the other hand, several potentially preventive strategies for sepsis following AKI exist (Table 2). It is worth mentioning that the evidence of clinical benefits is weak or even lacking in most of the strategies. First, supplement probiotics and short-chain fatty acid [65], or administer AST-120 (an oral adsorbent) [98] to protect against kidney injury and to decrease gut microbiota-derived metabolites which alter immune homeostasis. Second, supplement vitamin D for an immunomodulation effect. A low serum calcitriol level is associated with a lower survival rate in human sepsis [99]. Besides, vitamin D pretreatment attenuates renal oxidative stress in lipopolysaccharide-induced AKI by regulating antioxidant enzyme genes and blocking nuclear factor kappa B-mediated cell apoptosis [100]. Third, avoid fluid overload during the oliguric period in AKI. The management includes decreasing fluid administration and increasing fluid removal by diuretics or KRT. Fourth, avoid unnecessary catheter cannulation to lower the bloodstream infection risk. Fifth, for severe AKI patients with KRT indications, consider using hemofiltration and hemoadsorption to remove some cytokine and molecules that are harmful to immunity. For example, continuous hemofiltration with a polyacrylonitrile dialysis membrane (AN69ST) showed a high adsorption capacity and significant clearance to adsorb HMGB1 [101], and a hemoadsorption corrects high serum resistin levels in patients with septic shock in vitro and restores anti-bacterial neutrophil function [102].

## 6. Limitations and Further Prospects

Several limitations exist in determining the bidirectional pathophysiological mechanisms between AKI and sepsis. First, the causality association between sepsis and AKI is hard to establish since sepsis and AKI are multifactorial clinical entities with complex pathophysiologies, and the precise occurrence time points of sepsis and AKI are often vague. Second, the pathologic information on SA-AKI is limited because kidney biopsy might not be suitable for SA-AKI patients in their critical situations. The existing pathophysiology is mainly from animal experiments. Nonetheless, the Kidney Precision Medicine Project, a multicenter prospective cohort study aiming to evaluate human kidney tissue, might provide valuable data to unveil the mystery [103]. Third, the current AKI diagnosis is based on function biomarkers (serum creatinine and urine amount) that delay the timing of AKI diagnosis and fail to diagnose the specific etiology of AKI differentially. Some parameters indicating sepsis (e.g., sepsis-3 or other scores) or kidney injury (e.g., biomarkers of kidney tubular damage, measurement of renal blood flow, or other scores) might potentially aid value in defining SA-AKI after the validation with patients’ prognoses.

## 7. Conclusions

The current review provided a comprehensive review of the pathophysiological interplay between sepsis and AKI. The pathophysiological of SA-AKI primarily includes inflammatory cascade, macrovascular and microvascular dysfunction, and cell cycle arrest and apoptosis. On the other hand, the pathophysiology of sepsis following AKI contains fluid overload, hyperinflammatory state, immunosuppression, and KRT and catheter-associated infection. The preventive strategies for SA-AKI are non-specific, mainly focusing on infection control and preventing further kidney insults. However, the preventive strategies for sepsis following AKI might focus on decreasing some metabolites, cytokines, or molecules harmful to our immunity, supplementing vitamin D3 for its immunomodulation effect, and avoiding fluid overload and unnecessary catheter cannulation. Several limitations make a clear understanding of the bidirectional pathophysiologies difficult. The prospects to unveil the mystery and improve the prognoses of AKI patients contain conducting studies, such as the Kidney Precision Medicine Project, to investigate human kidney tissue and establish parameters or scores better to determine the occurrence timing of sepsis and AKI and the definition of SA-AKI.

## Figures and Tables

**Table 1:** Potentially preventive strategies for SA-AKI.

Strategies	Remarks
Prompt and proper antibiotics administration	Treatment of infection and sepsis
Vasopressor keeping a mean arterial blood pressure > 65 mmHg (norepinephrine preferred)	Maintain renal perfusion and autoregulation
Balanced crystalloid fluid administration	Avoidance of chloride overload. With benefit on major kidney adverse events
Avoidance of nephrotoxic agents	e.g., some antibiotics and contrast media
Application of the KDIGO bundle (?)	Effects under evaluation
High awareness of abdominal compartment syndrome	High intra- abdominal pressure is a deteriorating factor of SA-AKI

Abbreviations: SA-AKI, sepsis-associated acute kidney injury; KDIGO, Kidney Disease Improving Global Outcomes.

**Table 2:** Potentially preventive strategies for sepsis following AKI.

Strategies	Remarks
Probiotics and short chain fatty acid supplementation	Decrease gut-microbiota-derived metabolites and their immunosuppressive effects
AST-120 administration	
Vitamin D supplementation	Immunomodulation effect
Avoidance of fluid overload	Decrease tissue edema and infection risk
Avoidance of unnecessary catheter cannulation	Decrease bloodstream infection risk
KRT strategies	Hemofiltration and hemoadsorption for removing some cytokines and molecules that are harmful to immunity

Abbreviations: KRT, kidney replacement therapy.

## Data Availability

Not applicable.
